# *Mammillaria* Species—Polyphenols Studies and Anti-Cancer, Anti-Oxidant, and Anti-Bacterial Activities

**DOI:** 10.3390/molecules25010131

**Published:** 2019-12-29

**Authors:** Hosam O. Elansary, Agnieszka Szopa, Marta Klimek-Szczykutowicz, Karolina Jafernik, Halina Ekiert, Eman A. Mahmoud, Ahmed Abdelmoneim Barakat, Diaa O. El-Ansary

**Affiliations:** 1 Plant Production Department, College of Food and Agricultural Sciences, King Saud University, P.O. Box 2455, Riyadh 11451, Saudi Arabia; 2 Floriculture, Ornamental Horticulture, and Garden Design Department, Faculty of Agriculture (El-Shatby), Alexandria University, Alexandria 21527, Egypt; 3 Department of Geography, Environmental Management, and Energy Studies, University of Johannesburg, APK Campus, Johannesburg 2006, South Africa; 4 Department of Pharmaceutical Botany, Medical College, Jagiellonian University, ul. Medyczna 9, 30-688 Kraków, Poland; a.szopa@uj.edu.pl (A.S.); marta.klimek-szczykutowicz@doctoral.uj.edu.pl (M.K.-S.); karolina.jafernik@doctoral.uj.edu.pl (K.J.);; 5 Department of Food Industries, Damietta University, Damietta 34511, Egypt; emanmail2005@yahoo.com; 6 Botanical Gardens Research Department, Horticultural Research Institute (ARC), Alexandria 12311, Egypt; cactai2000@gmail.com; 7 Precision Agriculture Laboratory, Department of Pomology, Faculty of Agriculture (El-Shatby), Alexandria University, Alexandria 21527, Egypt; diaaagri@hotmail.com

**Keywords:** *Mammillaria*, stem extract, phenolic acids, anti-cancer, anti-oxidant, anti-bacterial, cytotoxicity

## Abstract

Discovering new natural resources of polyphenols is the aim of many recent studies in the field of natural product research. This study tentatively investigated the polyphenols profile of the stems of seven *Mammillaria* species *(M. rhodantha, M. spinosissima, M. hahniana, M. crucigera, M. candida, M. albilanata*, and *M. muehlenpfordtii*) using high performance liquid chromatography with DAD detector (HPLC-DAD) method. Furthermore, the anti-cancer, anti-oxidant, and anti-bacterial potentials of these extracts as well as major identified phenols were explored. The HPLC-DAD study confirmed the availability of six phenolic acids, including gentisic acid, chlorogenic acid, caffeic acid, protocatechuic acid, sinapic acid, and p-hydroxybenzoic acid. The dominant compounds were: gentisic acid in *M. rhodantha* and *M. spinosissima*; chlorogenic acid in *M. muehlenpfordtii, M. crucigera*, and *M. rhodantha;* and caffeic acid in *M. rhodantha, M. crucigera,* and *M. spinosissima.* Stems of *Mammillaria* sp. showed antiproliferative effects against HeLa, MCF-7, and Jurkat cells. In HeLa and MCF-7 cells, the best antiproliferative activities were found in the treatments with *M. rhodantha, M. spinosissima*, and *M. muehlenpfordtii*. The apoptotic assay of *M. rhodantha, M. spinosissima*, and *M. muehlenpfordtii* showed accumulation of necrotic cells in the early and late apoptotic phase. *M. rhodantha, M. spinosissima*, and *M. muehlenpfordtii* showed the highest anti-oxidant activities using 2,2-diphenyl-1-picrylhydrazyl (DPPH), β-carotene bleaching, and ferric reducing anti-oxidant power (FRAP) assays. *M. rhodantha* was the best source of antioxidants. *Mammillaria* sp. showed moderate anti-bacterial effects against bacteria and the highest effects were found using the extracts of *M. rhodantha, M. spinosissima, M. crucigera* and *M. muehlenpfordtii* against most bacteria. The anti-bacterial activities were attributed to other phenolic compounds (e.g., chlorogenic acid) than gentisic acid, which was not active against most bacteria. *Mammillaria* sp. could be considered to be an important natural source of phenolic acids with anti-cancer, anti-bacterial, and anti-oxidant activities.

## 1. Introduction

Polyphenols are commonly found in cereals, fruit, vegetables, and beverages. However, other resources are being explored such as tree barks [1,2] and fungi [3]. Polyphenols play an important role in controlling several diseases including cancer, diabetes, cardiovascular diseases, osteoporosis, neurodegenerative diseases, hypertension, and asthma, and act as antiaging compounds [4]. The application of polyphenols as anti-cancer is mainly attributed to their protective effects and they act as inhibitors for tumor growth and dissemination. The mechanism of action of polyphenols on tumors include antiproliferation, apoptosis, stimulating cell cycle arrest, anti-oxidant mechanism, and induction of detoxification enzymes, molecular regulation of cancer related genes, and anti-inflammatory activity [5,6,7]. One of the most important groups of plant polyphenols are flavonoids and phenolic acids. Some phenolic acids are recognized as strong anti-oxidant, anti-cancer, and antimicrobial metabolites [2,8,9,10,11,12]. 

Discovering new resources of antioxidants is one of the main objectives of natural product research investigations worldwide. Consumption of polyphenols is strongly associated with reduced levels of lymphocytic DNA oxidative damage; they protect cells and limit the risk of associated degenerative diseases. The anti-cancer effects of polyphenols are closely associated with its anti-oxidant activity. Polyphenols inhibit protein kinase C, cyclooxygenase, hydroperoxidase, Akt, focal adhesion kinase, NFκB, and Bcl-2 phosphorylation [1,13,14,15,16]. Polyphenols as plant secondary metabolites play a pivotal role in control bacterial infections both in plants and humans [17,18,19]. They show strong antimicrobial activities on human pathogenic bacteria by applying several mechanisms including cytoplasmic membrane destabilization, increasing cell membrane permeability, inhibiting microbial enzymes, influencing the metabolism of bacteria, and deprivation of essential minerals [20]. 

The *Mammillaria* genus is the largest in its family (Cactaceae), and contains more than 200 species of globose or ball-shaped cacti that carry small spines and flowers. These plants are native to the Western Hemisphere and especially Mexico. The family Cactaceae has some medicinal genera such as *Opuntia* sp. which are used as food and has important economic value as horticultural crop [21]. *Mammillaria* sp. are commonly used for the decoration and ornamenting gardens all over the world. However, no studies revealed the phenolic composition and biological activities of the extracts of any species of *Mammillaria*. Common species such as *M. rhodantha, M. spinosissima, M. hahniana, M. crucigera, M. candida, M. albilanata,* and *M. muehlenpfordtii* are widely used in spring flower festival decoration in cactus or desert gardens. They are decorated with plastic red and yellow synthetic flowers, because they rarely flower under the natural conditions which usually do not simulate their natural habitat. However, their natural flowers have different colors and are borne in rings around the column. The polyphenolic profile and biological activities of this genus has not been studied before. 

The *Mammillaria* genus is the most common genus used in the Cactaceae family in ornamenting gardens; however, experimental studies regarding the bioactivity of stem extracts is limited. In this study, the polyphenol profiles (aimed on chosen flavonoids and phenolic acids) of seven *Mammillaria* cacti were evaluated qualitatively and quantitatively using HPLC-DAD method for the first time. Furthermore, the anti-cancer, anti-oxidant, and anti-bacterial properties were explored. 

## 2. Results

### 2.1. Polyphenol Profiling of Mammillaria 

**Figure 1:**
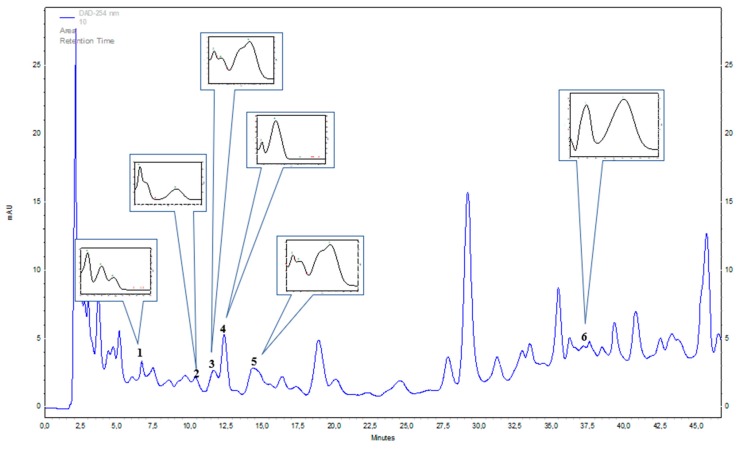
Representative HPLC-DAD (λ = 254 nm) chromatogram of *Mammillaria pringlei* stem extracts: 1—protocatechuic acid, 2—gentisic acid, 3—chlorogenic acid, 4—p-hydroxybenzoic acid, 5—caffeic acid, 6—sinapic acid.

Seven species from *Mammillaria* genus—*M. rhodantha, M. spinosissima, M. hahniana, M. crucigera, M. candida, M. albilanata*, and *M. muehlenpfordtii*—were tested tentatively with HPLC-DAD method to detect chosen phenolic acids and flavonoids. In the stem extracts, seven phenolic acids were identified: gentisic acid, chlorogenic acid, caffeic acid, protocatechuic acid, sinapic acid, and p-hydroxybenzoic acid (Table 1 and Figure 1) out of the 21 screened. None of 14 tested flavonoids were confirmed. The dominant compounds were: gentisic acid in *M. spinosissima* (40.44 mg 100 g^−1^ DW) and *M. rhodantha* (38.27 mg 100 g^−1^ DW); chlorogenic acid in *M. muehlenpfordtii* (30.88 mg 100 g^−1^ DW)*, M. crucigera* (14.61 mg 100 g^−1^ DW), *M. candida* (11.61 mg 100 g^−1^ DW) and *M. rhodantha* (10.37 mg 100 g^−1^ DW)*;* and caffeic acid in *M. rhodantha* (15.80 mg 100g^−1^ DW)*, M. crucigera* (7.17 mg 100 g^−1^ DW)*,* and *M. spinosissima* (9.66 mg 100 g^−1^ DW). Protocatechuic acid, sinapic acid, and p-hydroxybenzoic acid were estimated in relatively lower amounts in all studied species. Out of the studied *Mammillaria* species, *M. rhodantha* and *M. spinosissima* could be considered to be the richest source of the studied phenolic acids (Table 1 and Figure 1).

### 2.2. Anti-Cancer Effects 

**Figure 2:**
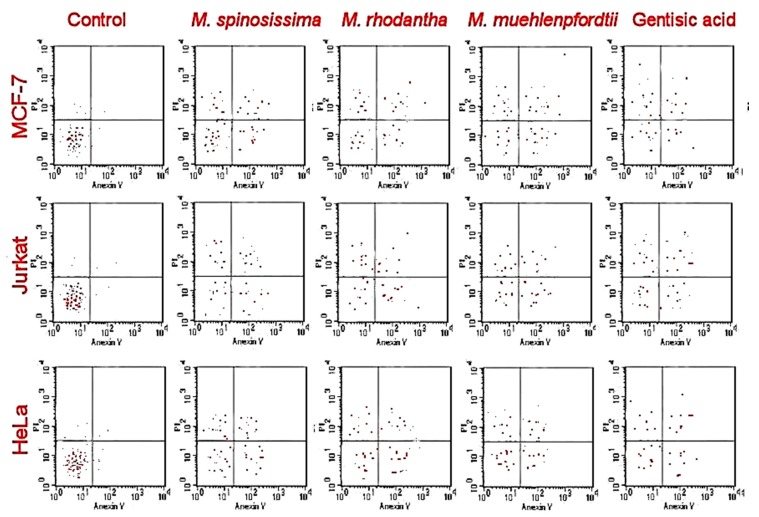
Apoptotic cell population (IC_50_) using flow cytometry.

Stems of *Mammillaria* sp. showed antiproliferative effects against selected cancer cells as shown in Table 2. The highest activities were against HeLa, MCF-7and Jurkat cells. In HeLa and MCF-7 cells, the best antiproliferative activities were found in the treatments with *M. rhodantha, M. spinosissima*, and *M. muehlenpfordtii*. The only anti-cancer activity against HT-29 was found in the extracts of *M. rhodantha* and *M. spinosissima*. The best anti-cancer effects against Jurkat were found in the treatments of *M. rhodantha* and *M. spinosissima*. Gentisic acid showed comparable activities to those of *M. rhodantha* and *M. spinosissima.* The apoptotic assay of *M. rhodantha, M. spinosissima*, and *M. muehlenpfordtii* showed accumulation of necrotic cells in the early and late apoptotic (Figure 2).

### 2.3. Anti-oxidant Effects 

*Mammillaria* sp. showed obvious anti-oxidant effects by several methods (Table 3). *M. rhodantha, M. spinosissima*, and *M. muehlenpfordtii* showed the highest anti-oxidant activities using DPPH, β-carotene bleaching, and ferric reducing anti-oxidant power (FRAP) assays compared to other species. *M. rhodantha* was best the source of antioxidants as revealed by lowest IC_50_. Furthermore, butylated hydroxytoluene (BHT) and Trolox showed higher activities than all stem extracts. 

### 2.4. Anti-Bacterial Activities 

*Mammillaria* sp. showed moderate anti-bacterial effects against all studied bacteria: *Bacillus cereus, Listeria monocytogenes, Escherichia coli, Micrococcus flavus, Staphylococcus aureus, and Pseudomonas aeruginosa* (Table 4). The highest effects were found using the extracts of *M. rhodantha, M. spinosissima, M. crucigera*, and *M. muehlenpfordtii* against most bacteria. Other *Mammillaria* sp. showed much lower anti-bacterial effects. The gentisic acid did not show activities against most bacteria. The chlorogenic acid showed high anti-bacterial effects which is comparable to antibiotics. 

## 3. Discussion

The seven *Mammillaria* species—*M. rhodantha, M. spinosissima, M. hahniana, M. crucigera, M. candida, M. albilanata*, and *M. muehlenpfordtii*—under HPLC-DAD qualitative and quantitative study showed that six phenols were present in all the species, which are gentisic acid, chlorogenic acid, caffeic acid, protocatechuic acid, sinapic acid, and p-hydroxybenzoic acid. This is the first report on the tentative phenolic composition of the stems of this genus. Indeed, further analyses with more chromatographic techniques would be recommended, for proper confirmation of detected compounds. Previous investigation revealed that the pollen of *Mammillaria heyderi* Sensu Lato contained quercetin, herbacetin glycoside derivatives, and kaempferol [22]. Under our study we did not detect quercetin or kaempferol in the studied steam extracts. The major phenol in *M. rhodantha* and *M. spinosissima* was gentisic acid. Gentisic acid was discovered in a few species such as *Momordica charantia* leaves (12.75 mg g^−1^) [23] and *Vaccinium oxyccocos* fruit (0.3 mg 100 g^−1^) [24] and is rare in general. Gentisic acid is a strong anti-oxidant and has important health benefits [25,26]. Chlorogenic acid is the ester of caffeic acid and is much more common than gentisic acid. Chlorogenic acid was isolated from different plants species such as *Etlingera elatior* leaves, coffee, and fruit [27] as well as medicinal plants [17]. The chlorogenic acid is known for strong its anti-oxidant, anti-cancer, anti-inflammatory, and antiviral properties, and modulates the anti-oxidant enzyme activities [28,29]. Protocatechuic acid, sinapic acid and p-hydroxybenzoic acid were relatively low but they are considered to be important anti-oxidant and anti-cancer compounds [30,31,32]. 

*Mammillaria* species showed antiproliferative effects against cancer cells. Most activities were against HeLa and MCF-7 cells using *M. rhodantha, M. spinosissima*, and *M. muehlenpfordtii* stem extracts. The three species are rich in specific phenolic compounds such gentisic acid, which is dominant in *M. rhodantha* and *M. spinosissima*, and chlorogenic acid, which is dominant in *M. muehlenpfordtii*. Gentisic acid showed comparable antiproliferative and cytotoxic activities to those of *M. rhodantha* and *M. spinosissima*. Gentisic acid is a quinonoid phenolic acid that is important for cancer prevention and treatment. These phenols activate the brain chemotherapeutics that help in the reduction of brain tumors [33] and has anti-oxidant activities related to the control of HCT-116 cancer cells [34]. In *M. muehlenpfordtii* there were strong antiproliferative activities against different cancer cells, which is explained by the activity of the major phenol found, which is chlorogenic acid. This phenol has anti-cancer activities on liver, colorectal, and laryngeal cancer. It influences the expression of specific genes such as *GSK-3β* and *APC* (up-regulation) and *β-catenin* (down-regulation) which promote the apoptosis of tumor cells [35]. The apoptotic activity of protocatechuic acid has been reported on human gastric carcinoma [36]. Sinapic and caffeic acids have been related to anti-cancer activities. Sinapic acid is considered to be a hydroxycinnamic acid derivative which up- and down-regulate specific genes in PC-3 and LNCaP prostate cancer cells [37]. Caffeic acid has anti-cancer activities against different cancer cells, such as human cervical cancer [38]. 

*Mammillaria* sp. had anti-oxidant activities as found in *M. rhodantha, M. spinosissima*, and *M. muehlenpfordtii* extracts. *M. rhodantha* was the best source of antioxidants in this study because it was rich in the strong antioxidants of gentisic acid, chlorogenic acid, caffeic acid, and protocatechoic acid. The four of these phenols have been associated with anti-oxidant activities as revealed in several studies [17,38,39]. Previous reports have found that gentisic acid provided protection to human erythrocytes against gamma radiation [25]. In another study, gentisic acid and protochatechuic acid imparted oxidative stability in sardine oil [32]. *M. spinosissima* is rich in gentisic acid and caffeic acid and both are strong antioxidants. *M. muehlenpfordtii* is rich in chlorogenic acid, which is responsible for anti-oxidant activities in this species. Previous investigation showed that chlorogenic acid can mitigate oxidative stresses in vitro and in vivo [39]. It was obvious that these dominant phenols were responsible for anti-oxidant activities. 

*Mammillaria* sp. showed anti-bacterial effects against bacteria, and the highest effects were found in the extracts of *M. rhodantha, M. spinosissima, M. crucigera* and *M. muehlenpfordtii* against most bacteria. The gentisic acid did not show activities against most bacteria. Gentisic acid showed weak or no activity against *E. coli, P. aeruginosa*, and *S. aureus* in the previous study [40]. The anti-bacterial activities of *M. rhodantha* is mainly attributed to other major phenols including chlorogenic acid, caffeic acid, and protocatechoic acid. Chlorogenic acid has anti-bacterial activities against bacteria such as *E. coli* [41] as found in the current study. It increases plasma membrane permeability, causing loss of barrier function [42]. Caffeic acid has antimicrobial activities against several microorganisms including *S. aureus* [43]. In a previous investigation, protocatechoic acid showed strong anti-bacterial activities against *E. coli, L. monocytogenes, S. aureus*, and *B. cereus* [44]. Furthermore, it has anti-oxidant, anti-inflammatory, and anti-cancer activities [30]. It was obvious that the anti-bacterial activities of the extracts of *M. rhodantha, M. spinosissima, M. crucigera*, and *M. muehlenpfordtii* against most bacteria was attributed to the chlorogenic composition as well as other major phenols. 

## 4. Materials and Methods 

### 4.1. Plant Material and Preparation

The stem of *Mammillaria* species (*M. rhodantha* subsp. *pringlei* (J.M. Coult.) D.R. Hunt *M. spinosissima* Lem., *M. hahniana* Werderm., *M. crucigera* Mart., *M. candida* Scheidw., *M. albilanata* Backeb., and *M. muehlenpfordtii*) were sampled from cactus nurseries in Alexandria, Egypt. Each species was identified by Ahmed Abdelmoneium and Hosam Elansary. Samples were vouchered at Alexandria (Hosam0001030–1037). Stems (3 replications) were dried using lyophilization (freeze dryers Labconco, Kansas City, MO, USA), then powdered [1,2]. The powder (0.2 g DW, dry weight), was put in 15 mL tubes, then subjected to extraction with methanol (10 mL, Chempur, Poland) by sonication (2 × 30 min at 30 °C) in an ultrasonic bath (Sonic-2, POLSONIC). Stem extracts were filtered using Whatman paper and left in crystallizers to evaporate methanol at room temperature. The residue was dissolved in methanol (1 mL, Merck for liquid chromatography LiChrosolv^®^, Merck KGaA, Darmstadt, Germany). The samples were maintained in deep freeze (−80 °C). A rotary evaporator was used to eliminate the methanol for bioassays. The bacterial and fungal cultures were obtained from the Faculty of Agriculture, Alexandria, Egypt. Cell cultures of breast adenocarcinoma (MCF-7), cervical adenocarcinoma (HeLa), T-cell lymphoblast (Jurkat), colon adenocarcinoma (HT-29), and HEK-293 (human normal cells). Cells were purchased from American-Type Culture Collection (ATCC). 

### 4.2. Chemicals

The following standards were used for phenolic acid qualification and quantification: benzoic acid and its derivatives (3,4-dihydroxyphenylacetic, ellagic, gallic, gentisic, *p*-hydroxybenzoic, protocatechuic, salicylic, syringic, and vanillic acids); cinnamic acid and its derivatives (caffeic, *o*-coumaric, *m*-coumaric, *p*-coumaric, ferulic, hydrocaffeic, isoferulic, and sinapic acids); and depsides (chlorogenic, neochlorogenic, and rosmarinic acids). To quantify the flavonoids, aglycone (kaempferol, luteolin, myricetin, quercetin and rhamnetin) and glycoside (apigetrin, cynaroside, hyperoside, isoquercetin, quercitrin, robinin, rutoside, trifolin, vitexin) standards were used. All the substances were acquired from Sigma–Aldrich, Germany.

### 4.3. Analyses of Phenolic Compounds

The stem extracts were studied by HPLC-DAD method [1,2,45,46] using the Merck-Hitachi liquid chromatograph (LaChrom Elite) with a DAD detector (L-2455) in a ultraviolet (UV)—visible spectra range of 200–400 nm (detection wavelength for all compounds was set at 254 nm). The Purospher RP-18e (250 × 4 mm; 5 μm, Merck) column was used and the temperature was set at 25 °C. The mobile phase consisted of A—methanol, B—methanol: 0.5% acetic acid 1:4 (*v/v*). The gradient was as follows: 100% B for 0–20 min; 100%–80% B for 20–35 min; 80%–60% B for 35–55 min; 60%–0% B for 55–70 min; 0% B for 70–75 min; 0%–100% B for 75–80 min; 100% B for 80–90 min with a flow rate (1 mL min^−1^). The injection volume was 20 µL and the compounds of interest were detected at 254 nm. The applied HPLC method was previously validated by our group [45,46]. The tested parameters were the following: accuracy; precision at three levels of standard substance concentrations in solution, 50%, 100%, and 150%; linearity; limit of detection (LOD); and limit of quantification (LOQ) [45,46]. Identification of compounds was performed either by comparison with UV spectra and retention times of reference substances or using co-chromatography. The compounds were quantified using the calibration curves method [45,46,47]. 

### 4.4. Anti-Cancer Activities 

Antiproliferative and cytotoxic effects of stem extracts were tested on MCF-7, HeLa, Jurkat, and HT-29, as well as HEK-293 (human normal cells) [1,2,16]. Extracts were solubilized in DMSO (1%). Vinblastine sulfate and taxol were considered positive controls. A microplate reader (Thermo, Waltham, MA, USA) was used at a 570 nm wavelength. The percentage of activity inhibition was calculated in triplicate: 

% Inhibition = (Abs. 570 nm control‒Abs. 570 nm sample)/Abs. 570 nm control × 100. Furthermore, IC_50_ values were obtained by plotting the percentage of cell viability against extract concentration and expressed in µg/mL. The IC_30_ and IC_50_ were determined in the apoptotic cell population (flow cytometry, FAC Scan, Becton Dickinson, Iowa, USA) following [1,15,16]. Control cells were untreated with extracts or methanol standards. 

### 4.5. Anti-Oxidant Activity 

DPPH, β-carotene bleaching [48] and FRAP [2,49] assays were used. For DPPH, the wavelength of 517 nm was used to determine the absorbance. During the β-carotene bleaching assay, the wavelength of 470 nm was used. Samples amount required to scavenged 50% of the DPPH/β-carotene bleaching solutions (IC_50_ in µg/mL) was determined by plotting the inhibition percent against extract concentration. The butylated hydroxytoluene (BHT) was incorporated as control. The FRAP reagent was prepared as described in previous studies (e.g., [43]) using tripyridyl triazine (TPTZ, Sigma–Aldrich, Berlin, Germany). Aliquots (100 μL) of bark extracts or Trolox (Sigma–Aldrich, Berlin, Germany) were added to FRAP reagent (3 mL), mixed, incubated for half an hour at 37 °C and the absorbance was measured at 593 nm. Aqueous solutions of known serial concentrations of Trolox (0–0.5 Mmol/L) were used for the calibration. Two sets of triplicate replications were conducted for all experiments.

### 4.6. Anti-Bacterial Activity

stem extracts effects against *Bacillus cereus* (ATCC 14579), *Listeria monocytogenes* (clinical isolate)*, Escherichia coli* (ATCC 35210), *Micrococcus flavus* (ATCC 10240), *Staphylococcus aureus* (ATCC 6538), and *Pseudomonas aeruginosa* (ATCC 27853) using the micro-dilution method [17,50]. Microtiter plates (96-well) containing a serial concentration of the stem extracts (0.05–200 mg mL^−1^) in each well mixed with bacterial inoculum (1.0 × 10^4^ CFU per well) in 100 μL tryptic soy broth were incubated at 37 °C for 24 h in a rotary shaker. The minimum inhibitory concentration (MIC) was defined as the lowest concentration of the stem extract that exhibited no visible growth using a binocular microscope and was determined following the incubation period of the microtiter plates. The minimum bactericide concentration (MBC), which was defined as the lowest concentration that caused no visible growth and indicated the killing of 99.5% of the inoculum, was determined using serial sub-culturing of the stem extract or standard. The optical density was determined at a wavelength of 655 nm. A positive control was used (streptomycin, 0.01–10 mg/mL), as well as a negative one (DMSO, 1%). The optical density was determined at a wavelength of 655 nm. A positive control was used (streptomycin, 0.01–10 mg/mL), as well as a negative one (DMSO, 1%).

### 4.7. Statistical Analyses 

The SPSS (Version 21.0) was used to determine least significance difference (LSD). The standard deviation (SD) of means of three replicates was used. 

## 5. Conclusions

This is the first report exploring seven *Mammillaria* species giving information about phenolic acid composition, as well as anti-cancer, anti-oxidant, and anti-bacterial activities. In all studied species, gentisic, chlorogenic, caffeic, protocatechuic, sinapic, and p-hydroxybenzoic acids were estimated qualitatively and quantitatively. The dominant compounds were gentisic acid in *M. rhodantha* and *M. spinosissima*, chlorogenic acid in *M. muehlenpfordtii, M. crucigera*, and *M. rhodantha*, and caffeic acid in *M. rhodantha, M. crucigera,* and *M. spinosissima.* The quantitative dominant were confirmed as gentisic acid in *M. spinosissima* (40.44 mg 100g^−1^ DW) and chlorogenic acid in *M. muehlenpfordtii* (30.88 mg 100g^−1^ DW). Stem extracts of *Mammillaria* sp. showed antiproliferative effects against HeLa, MCF-7, and Jurkat cells. In HeLa and MCF-7 cells, the best antiproliferative activities were found in treatments with *M. rhodantha, M. spinosissima*, and *M. muehlenpfordtii*. The only anti-cancer activity against HT-29 was found in the extracts of *M. rhodantha* and *M. spinosissima*. The apoptotic assay of *M. rhodantha, M. spinosissima*, and *M. muehlenpfordtii* showed the accumulation of necrotic in the early and late apoptotic (Figure 2). *M. rhodantha, M. spinosissima*, and *M. muehlenpfordtii* showed the highest anti-oxidant activities using DPPH, β-carotene bleaching, and FRAP assays compared to other species. *M. rhodantha* was the best source of antioxidants as revealed by lowest IC_50_. *Mammillaria* sp. showed moderate anti-bacterial effects against bacteria, and the highest effects were found in the extracts of *M. rhodantha, M. spinosissima, M. crucigera*, and *M. muehlenpfordtii* against *B. cereus*, *L. monocytogenes, E. coli*, *M. flavus*, *S. aureus* and *P. aeruginosa.* Gentisic acid did not show activities against most bacteria. Chlorogenic acid showed high anti-bacterial effects, which was comparable to the antibiotic used. *Mammillaria* sp. could be considered to be a new natural resource of phenols with biological activities. 

## Figures and Tables

**Table 1:** The quantitative (mg 100 g^−1^ DW ± SD) estimations of phenolic acids in *Mammillaria* sp. stem extracts.

	Protocatechuic Acid	Gentisic Acid	Chlorogenic Acid	p-Hydroxy-Benzoic Acid	Caffeic Acid	Sinapic Acid
*M. rhodantha*	4.16 ± 0.44	38.24 ± 4.18	10.37 ± 1.06	2.64 ± 0.21	15.80 ± 1.67	3.07 ± 0.35
*M. spinosissima*	4.20 ± 0.40	40.44 ± 4.62	6.23 ± 0.63	0.96 ± 0.09	9.66 ± 0.94	7.88 ± 0.76
*M. hahniana*	3.90 ± 0.31	5.63 ± 0.58	8.61 ± 0.82	1.47 ± 0.14	6.38 ± 0.62	2.51 ± 0.31
*M. crucigera*	3.42 ± 0.28	7.63 ± 1.07	14.61 ± 1.11	0.86 ± 0.10	7.17 ± 0.58	6.99 ± 0.68
*M. candida*	1.54 ± 0.18	0.83 ± 0.09	11.61 ± 1.09	0.88 ± 0.10	0.35 ± 0.05	1.36 ± 0.03
*M. albilanata*	1.89 ± 0.23	1.00 ± 0.10	4.26 ± 0.50	0.52 ± 0.05	2.25 ± 0.26	2.58 ± 0.34
*M. muehlenpfordtii*	3.08 ± 0.32	1.81 ± 0.25	30.88 ± 3.51	3.80 ± 0.32	2.75 ± 0.22	4.25 ± 0.45

**Table 2:** Antiproliferative activity [IC_50_ (µg mL^−1^)] of *Mammillaria* sp. stem extracts (mg mL^−1^) as well as gentisic acid on cancer cells.

	HeLa	Jurkat	HT-29	MCF-7	HEK-293
Control	6.5 ± 0.1	22.0 ± 0.9	118.05 ± 5.7	21.94 ± 0.9	˃200
*M. rhodantha*	20.2 ± 0.2	18.3 ± 1.1	68.25 ± 3.3	28.9 ± 1.3	˃200
*M. spinosissima*	11.6 ± 0.3	10.6 ± 0.5	49.10 ± 2.6	22.3 ± 1.1	˃200
*M. hahniana*	168.57 ± 5.4	88.32 ± 3.3	˃200	56.1 ± 2.9	˃200
*M. crucigera*	173.18 ± 9.2	96.87 ± 5.2	˃200	64.9 ± 3.1	˃200
*M. candida*	195.35 ± 10.8	79.65 ± 3.7	˃200	57.25 ± 2.5	˃200
*M. albilanata*	163.22 ± 9.2	94.75 ± 4.7	˃200	63.25 ± 3.5	˃200
*M. muehlenpfordtii*	36.8 ± 1.2	26.17 ± 2.1	85.22 ± 0.2	33.85 ± 2.7	˃200
Gentisic acid	5.8 ± 0.3	7.9 ± 0.5	25.85 ± 1.3	10.85± 1.2	˃200
Vinblastine sulfate	2.1 ± 0.09	0.1 ± 0.02	19.32 ± 1.4	‒	47.1 ± 1.3
Taxol	‒	‒	‒	0.08 ± 0.008	‒

**Table 3:** DPPH and β-carotene bleaching acid of *Mammillaria* sp. stem extracts as well as gentisic acid.

	DPPH (IC_50_, µg mL^−1^)	β-Carotene-Bleaching Assay (IC_50_, µg mL^−1^)	FRAP (IC_50_, mM TEAC/g Extract)
*M. rhodantha*	9.2 ± 0.3c	11.1 ± 0.5c	13.6 ± 0.9e
*M. spinosissima*	7.3 ± 0.2cd	9.2 ± 0.1d	11.7 ± 0.7e
*M. hahniana*	20.9 ± 0.8a	26.0 ± 0.9a	34.9 ± 2.5a
*M. crucigera*	19.5 ± 0.7ab	24.1 ± 1.5a	31.2 ± 1.1b
*M. candida*	20.6 ± 1.7ab	25.9 ± 2.3a	32.7 ± 2.3ab
*M. albilanata*	18.4 ± 1.1b	21.5 ± 0.6b	26.9 ± 1.8c
*M. muehlenpfordtii*	10.8 ± 0.5c	12.8 ± 1.1c	15.3 ± 1.5d
Gentisic acid	5.9 ± 0.3d	7.5 ± 0.3d	10.1 ± 1.3f
BHT	2.8 ± 0.1e	3.2 ± 0.1e	—
Trolox	—	—	3.4 ± 0.1g

Values with different letters within a column indicates significant differences (*p* = 0.05). TEAC: Trolox equivalents anti-oxidant.

**Table 4:** Minimum inhibitory (MIC) and bactericidal concentration (MBC) of *Mammillaria sp.* stem extracts (mg mL^−1^) as well as gentisic and chlorogenic acids.

	*B. cereus* MIC MBC	*P. aeruginosa* MIC MBC	*L. monocytogenes* MIC MBC	*E. coli* MIC MBC	*M. flavus* MIC MBC	*S. aureus* MIC MBC
***ATCC***	14579	27853	clinical isolate	35210	10240	6538
***M. rhodantha***	0.12 ± 0.01	0.20 ± 0.02	0.27 ± 0.02	0.23 ± 0.03	0.31 ± 0.02	0.27 ± 0.01
	0.43 ± 0.03	0.57 ± 0.03	0.82 ± 0.04	0.49 ± 0.03	0.72 ± 0.04	0.73 ± 0.03
***M. spinosissima***	0.15 ± 0.01	0.27 ± 0.02	0.29 ± 0.01	0.24 ± 0.02	0.37± 0.01	0.29 ± 0.03
	0.49 ± 0.03	0.65 ± 0.03	0.95 ± 0.03	0.52 ± 0.03	0.88 ± 0.05	0.85 ± 0.05
***M. hahniana***	0.35 ± 0.02	0.64 ± 0.01	0.59 ± 0.01	0.49 ± 0.01	0.78 ± 0.03	2.21 ± 0.21
	0.83 ± 0.04	2.83 ± 0.16	1.78 ± 0.19	1.53 ± 0.12	2.48 ± 0.16	6.42 ± 0.65
***M. crucigera***	0.13 ± 0.01	0.23 ± 0.01	0.32 ± 0.03	0.27 ± 0.01	0.23 ± 0.01	0.42 ± 0.03
	0.37 ± 0.03	0.68± 0.09	1.12 ± 0.11	1.32 ± 0.05	0.65 ± 0.03	1.33 ± 0.11
***M. candida***	0.29 ± 0.01	0.89 ± 0.01	1.17 ± 0.02	0.56 ± 0.01	0.67 ± 0.03	1.98 ± 0.01
	0.87 ± 0.05	3.20 ± 0.15	3.92 ± 0.12	2.82 ± 0.31	2.23 ± 0.13	6.54 ± 0.53
***M. albilanata***	0.36 ± 0.02	1.10 ± 0.07	1.20 ± 0. 10	1.25 ± 0.02	2.14 ± 0.21	1.19 ± 0.01
	0.93 ± 0.03	5.56± 0.28	4.75 ± 0.21	4.74 ± 0.52	7.87 ± 0.54	3.38 ± 0.17
***M. muehlenpfordtii***	0.17 ± 0.01	0.29 ± 0.02	0.35 ± 0.01	0.43 ± 0.01	0.35± 0.02	0.29± 0.03
	0.48 ± 0.03	0.78 ± 0.03	1.14 ± 0.18	1.85 ± 0.18	0.91 ± 0.05	0.92 ± 0.03
**Gentisic acid**	N.D.	N.D.	N.D.	23.20 ± 0.02	N.D.	N.D.
	N.D.	N.D.	N.D.	> 100	N.D.	N.D.
**Chlorogenic acid**	0.13 ± 0.01	0.07 ± 0.01	0.15 ± 0.01	0.18 ± 0.03	0.21 ± 0.02	0.25± 0.01
	0.35 ± 0.02	0.34 ± 0.03	0.37± 0.03	0.45 ± 0.05	0.63 ± 0.03	0.63 ± 0.03
**Streptomycin**	0.08 ± 0.01	0.07 ± 0.01	0.13 ± 0.01	0.11 ± 0.01	0.10 ± 0.01	0.15 ± 0.01
	0.17 ± 0.01	0.15 ± 0.01	0.28 ± 0.03	0.23 ± 0.01	0.20 ± 0.02	0.32 ± 0.02

*Bacillus cereus* (ATCC 14579), *Listeria monocytogenes* (clinical isolate), *Escherichia coli* (ATCC 35210), *Micrococcus flavus* (ATCC 10240), *Staphylococcus aureus* (ATCC 6538), and *Pseudomonas aeruginosa* (ATCC 27853)

## Abbreviations

HPLC-DAD	High-Performance Liquid Chromatography with Diode-Array Detection
MCF-7	cell cultures of breast adenocarcinoma
HeLa	cell cultures of cervical adenocarcinoma
Jurkat	cell cultures of T-cell lymphoblast
HT-29	cell cultures of colon adenocarcinoma
ATCC	American-Type Culture Collection
